# The role of air pollution in myocardial remodeling

**DOI:** 10.1371/journal.pone.0176084

**Published:** 2017-04-20

**Authors:** A. M. de Oliveira-Fonoff, C. Mady, F. G. Pessoa, K. C. B. Fonseca, V. M. C. Salemi, F. Fernandes, P. H. N. Saldiva, F. J. A. Ramires

**Affiliations:** 1 Cardiomyopathy Unit at the Heart Institute (InCor) of the University of São Paulo, São Paulo, Brazil; 2 Department of Pathology, Medical School of the University of São Paulo, São Paulo, Brazil; The Ohio State University, UNITED STATES

## Abstract

**Background:**

Excessive air pollution in urban environments can impact morbidity and mortality. The authors evaluated the role of particulate matter_2.5_ (PM_2.5_) in structural, geometric, and functional remodeling in hearts, using an experimental model of myocardial infarction.

**Methods and findings:**

Seventy-five rats were divided into 5 groups: control (CG), CG exposed to PM_2.5_ pollution (CGP), myocardial infarcted group (MI), infarcted group immediately exposed to pollution (IGP-I), and infarcted group previously exposed to pollution and kept exposed after infarction (IGP-II). Greater deposition of interstitial collagen occurred in the left ventricle in CGP, MI, IGP-I, and IGP-II groups compared with that in controls (p = 0.002 CG vs CGP and p<0.0001 CG vs MI, IGP-I, and IGP-II). In the right ventricle, greater collagen deposition existed in CGP, MI, IGP-I, and IGP-II compared with that in CG (p<0.021 CG vs CGP and p<0.0001 CG vs MI, IGP-I, and IGP-II). At the end of the study, CG had a higher mean shortening fraction than the other groups had (p≤0.03). Left ventricular systolic diameter was lower in CG than in infarcted groups (p≤0.003). The infarcted groups had greater expression of TGF-β (p≤0.04). PM_2.5_ increased the expression of TGF-β in the IGP-II compared with the MI group (p = 0.004). The TNF-α gene was overexpressed in the IGP-II compared with the CGP group (p = 0.012). INF-γ gene expression was greater in IGP-II (p≤0.01). Oxidative stress analysis showed a higher glutathione concentration in CGP (p = 0.03), MI (p = 0.014), and IGP-I (p = 0.008) compared with that in CG.

**Conclusions:**

PM_2.5_ stimulates the deposition of fibrosis in the myocardium of healthy hearts, but not in infarcted hearts. PM_2.5_ modulates the inflammatory response, which was greater in the IGP-II group. It also modulates oxidative stress in healthy hearts but not in infarcted hearts.

## Introduction

According to the World Health Organization, in 2012 there were approximately 3.7 million global deaths associated with exposure to particulate matter [1]. Air pollution is a significant environmental issue in large cities with a high population density. Vehicular and industrial emissions are among the main causes of high levels of air pollution. In fact, there is a direct relationship between increased pollution and high morbidity and mortality rates, resulting in increased hospital admissions related to heart and lung diseases [2, 3]. The most harmful compound in pollution seems to be particulate matter (PM), regarding injury to the heart. Several studies have shown that the size of the PM and its surface area are decisive causes of inflammatory injury, oxidative damage, and other biological effects [4–6]. This damage is more extensive with fine (PM_2.5_) and ultrafine particles (PM_0.1_), which have a greater potential to penetrate into the respiratory tract and reach the lung alveoli. Moreover, the source of PM, regardless of its size, may have an impact on health. More than 50% of particulate materials are retained in pulmonary parenchyma, and a very small amount of particulate material might be taken into the circulatory stream. PM retained in the pulmonary parenchyma triggers a complex cascade of events, such as inflammation, oxidative stress, and autonomic impairment, which in turn spread this same cascade of events via different messengers throughout the entire body, including the heart. Particulate matter comes in a wide variety of sizes and components depending on several factors. The main components of PM are organic compounds, transition metal ions (sulfate and nitrate), quinoid stable radicals of carbonaceous materials, biological sources, minerals, and reactive gases. Toxicological research shows that particulate matter has numerous harmful mechanisms, such as cell cytotoxicity due to oxidative stress, oxidative damage to DNA, mutagenicity, and stimulation of inflammatory factors [7, 8]. Specifically, in the myocardium affected by an ischemic process, cardiomyocyte necrosis, apoptosis, activation of the complement system, and inflammatory cell accumulation are known to occur in the infarcted and remote areas. The pathways for this cardiomyocyte loss include inflammation and oxidative stress. This complex chain of events promotes intense molecular and cellular remodeling in the infarcted region and also in regions distant to it [9, 10]. Air pollution can be linked directly to cardiac remodeling in mediating oxidative stress and inflammation [11]. However, the available literature does not correlate exposure to pollution with myocardial remodeling, inflammation, and oxidative stress. So, because pollution can activate the chain of events including oxidative stress and inflammation, which are the same pathways activated by heart injury, our hypothesis is that air pollution might amplify heart remodeling after myocardial infarction.

The aim of this study was to evaluate the role of PM_2.5_ in structural, geometric, and functional remodeling in the heart by using an experimental model of myocardial infarction.

## Methods

### Animal model

We studied 75 male Wistar rats weighing 250–300 g that were supplied to us by the animal breeding facility at the University of São Paulo. These animals were divided into the following 5 groups with 15 animals in each: (1) control group (CG), (2) control group exposed to pollution (CGP), (3) myocardial infarcted group (MI), (4) infarcted group immediately exposed to pollution (IGP-I), and (5) infarcted previously exposed to pollution for 3 weeks and continued on pollution exposure after infarction for 4 additional weeks (IGP-II). MI was induced while the rats were anesthetized with ketamine and xylazine. The animals were intubated and put on mechanical ventilation. The chest was then opened and the left coronary artery ligated. All efforts were made to minimize animal suffering. During the immediate postoperative period and until the second day, we administered Tramadol (40mg/kg) twice a day by intraperitoneal infusion to minimize pain, according to the institutional protocol.

The protocol was performed in accordance with the recommendations of COBEA (the Brazilian College of Experimental Animal Studies) [12] and the Guide for the Care and Use of Laboratory Animals (UFAW) [13]. The study protocol was reviewed and approved by the ethics committee (CEUA: 335/10) of the Heart Institute and the University of São Paulo Medical School. The animals’ health was monitored daily. Loss of mobility, weight, and pelage were criteria used to evaluate for illness.

The animals were euthanized after 4 weeks of MI using intracardiac 3 M potassium chloride while the rats were anesthetized with ketamine and xylazine. The heart was removed through a median sternotomy, weighed, and then divided into 3 parts: (1) the apex was frozen in liquid nitrogen and stored at -80°C, excluding the infarcted area; (2) the middle was cut at the largest MI area macroscopically identified and fixed in 10% formalin, for histology; and (3) the base was also frozen in liquid nitrogen and stored at -80°C. A blinded technician collected all hearts.

There was 10% mortality during the establishment of the MI model. Deceased animals were replaced before the protocol started. After the beginning of the protocol, no deaths occurred. The animals with a less than 10% MI size were excluded. Therefore, the study groups were CG = 15, CGP = 15, MI = 8, IGP-I = 11, and IGP-II = 11. No animal was excluded because of sickness.

### Exposure protocol

**Fig 1:**
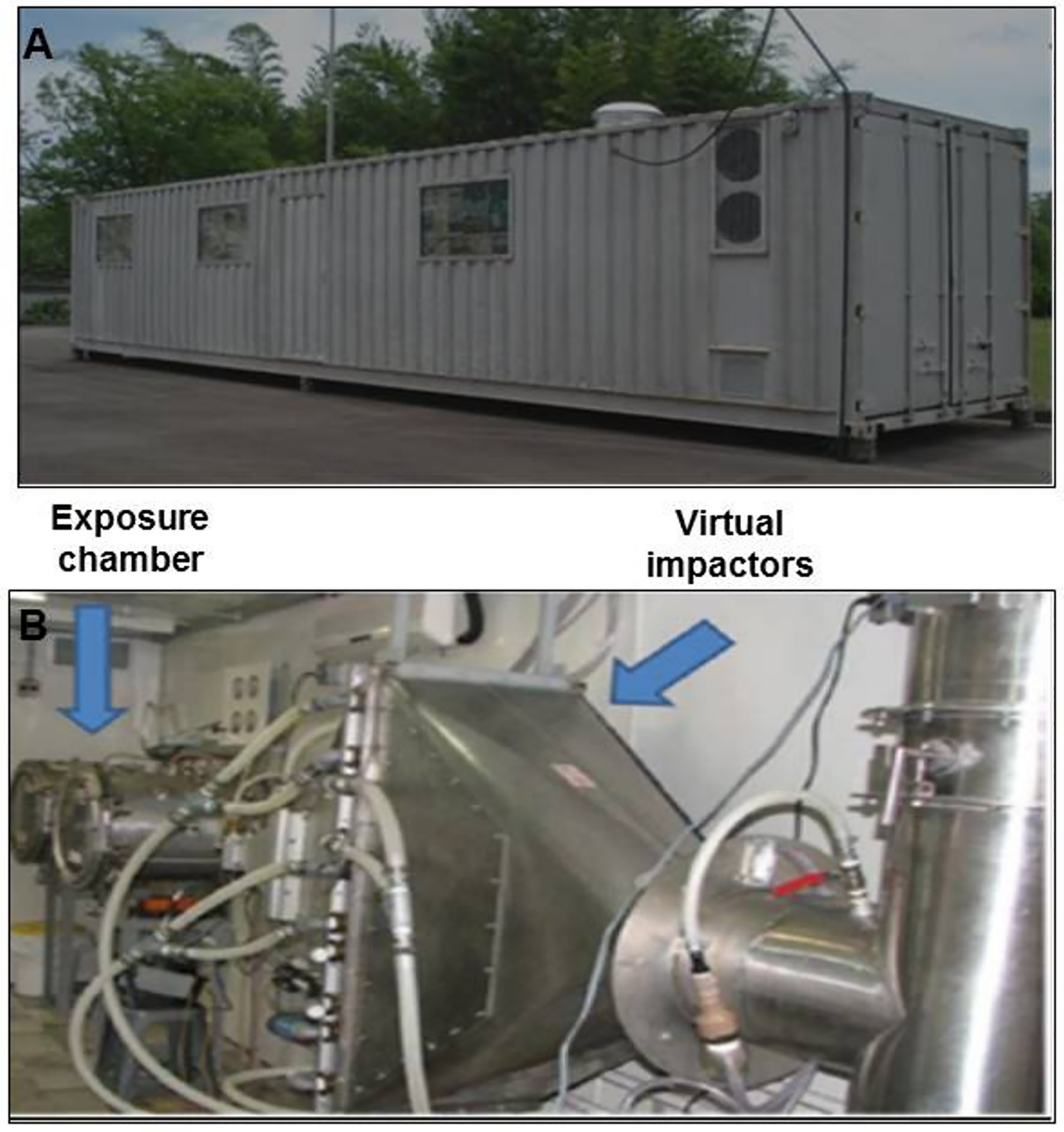
**A:** Photograph of container where the air pollution was captured from the fleet of vehicles. **B:** Internal space of the container with the exposure chambers.

The technique developed by Davel et al. was used to create local exposure to PM (PM_2.5_) [14]. A container was placed beside a very high traffic avenue with an average of 85,000 vehicles per day (diesel and gasoline). The gases emitted by this fleet of vehicles were collected into this container. The chamber system has serial filters that remove large particles, and by using a Harvard Ambient Particle Concentrator (HAPC), it selects PM. In this system, a jet of particle-laden air is injected and a series of impactors is used to classify particles according to their aerodynamic size [14] (Fig 1). The particle composition was determined by using an X-ray fluorescence analyzer (Table 1). The CGP and IGP-I animals received a concentration of PM_2.5_ at a daily average of between 600–800 μg/m^3^, 5 days a week, for 4 weeks, or 7 weeks in the IGP-II group (3 weeks before and 4 weeks after MI).

**Table 1:** Particle composition.

**PM**_2.5_*	19.78 ± 11.03
Black Carbon*	6.65 ± 6.43
**Na**	200.91 ± 166.19
**Mg**	0.18 ±0.73
**Al**	90.52 ± 81.06
**Si**	361.37 ± 200.37
**P**	33.49 ± 25.74
**S**	1441.00 ± 958.67
**Cl**	25.03 ± 28.70
**K**	354.86 ± 346.30
**Ca**	97.22 ± 50.63
**Ti**	9.26 ± 7.23
**V**	0.85 ± 1.19
**Cr**	0.33 ± 0.65
**Mn**	4.94 ± 2.90
**Fe**	182.92 ± 113.97
**Ni**	0.68 ± 0.63
**Cu**	6.55 ± 7.20
**Zn**	66.58 ± 43.33
**Se**	2.52 ± 2.96
**Br**	6.81 ± 7.80
**Pb**	9.67 ± 5.93

All concentration are depicted as mean ± SD and in nanograms per cubic meter, except where otherwise noted.

*concentration in micrograms per cubic meter.

### Collagen morphometry

Serial paraffin sections (4 μm) of the formalin-fixed hearts were stained with fibrillar collagen-specific Picrosirius red. Stained coronal sections from both ventricles were then viewed with light microscopy using x10 objective to identify sites of fibrosis, including those in the interstitial and perivascular spaces. The interstitial collagen volume fraction (ICVF) for both ventricles was determined by video morphometry, separately and excluding the infarcted area, using Leica QWIN image processing and analysis software (Leica Microsystems, Cambridge, United Kingdom Ltd) [15]. We randomly analyzed 2 sections of each heart from every animal in all groups. All examiners were blinded for the groups.

### Infarct size

**Fig 2:**
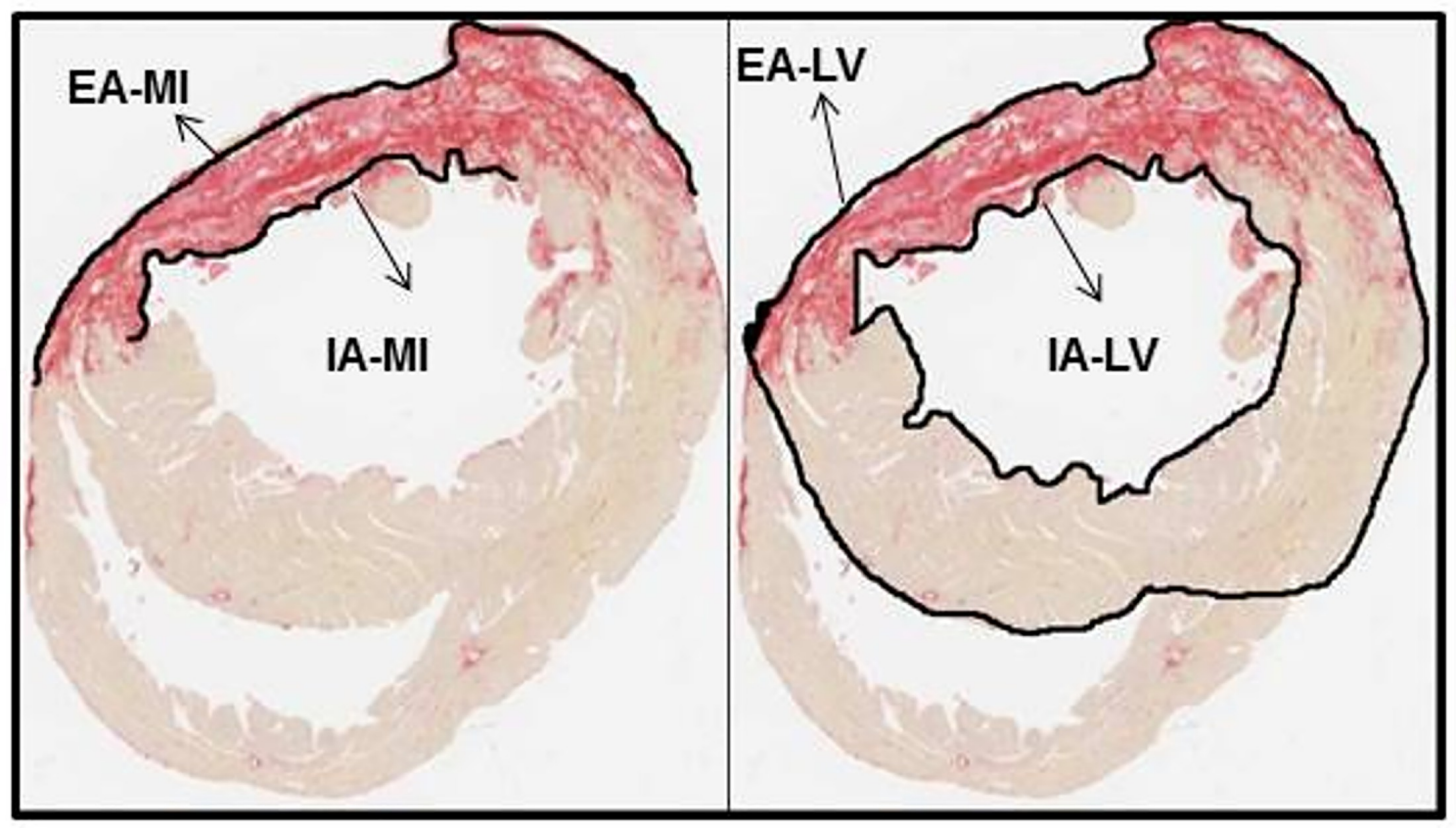
Infarct size. Coronal sections of the whole heart stained by Picrosirius red. EA-MI = external area of myocardial infarction, IA-MI = internal area of myocardial infarction, EA-LV = external area of left ventricle, IA-LV = internal area of left ventricle.

The same sections used for the quantification of collagen were used to determine infarct size. Blinded investigators examined one section obtained from the largest surface length of the infarcted left ventricular wall of each infarcted animal. By using the image analysis processor QWIN Leica Image software (Leica Microsystems, Cambridge Ltd.), cuts of the ventricular circumference encompassing the entire left ventricle of each sample were photographed. The MI size was defined as the region corresponding to the ventricular wall between the outer edges where scar tissue was identified. We measured the circumferences of the infarcted and noninfarcted areas (Fig 2). The size of the infarcted area was then calculated using the formula below as the percentage of the length of the MI in relation to the whole left ventricular circumference of each section [15]. Infarct size (%) = {[(EAMI + IAMI) / 2] / [(EALV + IALV) / 2]} x100 (EA = external perimeter; IA = internal perimeter; LV = left ventricle; MI = myocardial infarction)

### Histology

For the semi-quantitative inflammatory process, we analyzed leukocyte/ macrophage infiltration of myocardial tissue. Using hematoxylin and eosin-stained hearts under microscopy with Leica QWIN image processing and analysis software (Leica Microsystems, Cambridge, United Kingdom Ltd), we counted the cells and established a ratio between number of cells and myocardial area analyzed (cell/mm^2^). We randomly analyzed one section for each animal using x10 objective, which resulted in a mean of 54.66 ± 2.51 fields for each section.

### Echocardiography

Transthoracic M-mode, 2-dimensional, pulsed Doppler echocardiography was performed using an Acuson, Sequoia model 512 (Siemens Healthcare—Erlangen, Germany), 9-mm transducer with a frequency of 13 MHz. The patterns of regional and global contraction were evaluated in real time in the parasternal long and short axes of the left ventricle (LV). The cardiac systolic and diastolic dimensions were assessed using the M mode. Left ventricular function was evaluated by fraction shortening analysis. The animals underwent echocardiography at the beginning and end of the study after being anesthetized with a combination of ketamine (50 mg/kg) and xylazine (10 mg/kg) administered intraperitoneally. The test was performed according to its standardization for rodents [16], and the echocardiograms of healthy animals in the control group were considered normal.

### Quantitative real-time qPCR analysis for inflammation, apoptosis, and ventricular overload

For gene expression analysis, we used the heart apex from the MI-excluded infarction zone. After removing the section, all samples were snap-frozen in liquid nitrogen. Following total RNA isolation, including DNase treatment with Turbo DNA-Free^®^ (Ambion—The RNA Company), RNA was reverse transcribed using SuperScript^®^ II Reverse Transcriptase (Invitrogen^®^). Quantitative real time-PCR was performed with the StepOnePlus^®^ Real-Time PCR System (Applied Biosystems, EUA) using the SYBR green method. The primers used for housekeeping were GAPDH (glyceraldehyde-3-phosphate dehydrogenase) (Gene Bank NM_017008) and Beta Actin (Gene Bank NM_031144). The following were also used: BNP (brain natriuretic peptide) (Gene BankTM NM_031545) for ventricular overload; Bcl-2 (B-cell lymphoma 2) (Gene BankTM NM_016993) and p53 (tumor protein p53) (Gene BankTM NM_030989) for apoptosis; TNF-α (tumor necrosis factor—alpha) (Gene BankTM NM_012675.3), TGF-β1 (transforming growth factor—beta 1) (Gene BankTM NM_021578.2), IL-1β (Interleukin 1 beta) (Gene BankTM NM_031515.2), IL-6 (Interleukin 6) (Gene BankTM NM_012589.1), INF-gamma (Interferon gamma) (Gene BankTM NM_138880.2), CCl-3 (chemokine [C-C motif] ligand 3) (Gene BankTM NM_013025), CCl-21 (chemokine [C-C motif] ligand 21) (Gene BankTM NM_001008513.1), and CCr-7 (chemokine [C-C motif] receptor 7) (Gene BankTM NM_199489.3) for inflammation.

### Oxidative stress

We used 2 commercial kits to evaluate oxidative stress: the ADMA (asymmetric dimethylarginine) direct (mouse/rat) ELISA (enzyme-linked immunosorbent assay) kit (Enzo Life Sciences^®^) [17] for serum quantification and the DetectX^®^ Glutathione (GSH) Fluorescent Detection Kit (Arbor Assays^®^) [18] for plasma. The quantification of the ADMA concentration was performed with 100 μL of serum in 96-Well Microplates and read at 450 nm. Briefly, serum was prepared in 5% sulphosalicylic acid and centrifuged at 14,000 g 4°C for 10 min to separate the proteins. Afterwards, supernatants were used to measure total GSH. The reaction was performed in 96-Well Microplates for fluorescence. Fluorescence readings were performed using a fluorescent emission of 510 nm. For both kits, the manufacturer's instructions were followed.

### Statistics

We used a nonparametric Kruskal-Wallis test to compare all measures between the 4 groups and the Dunn test for the differences [19]. The level of significance was 5%. Therefore, the description of the level of the test (p value) was less than 0.05. The data are presented as mean ± standard deviation.

## Results

### Histological analyses

**Fig 3:**
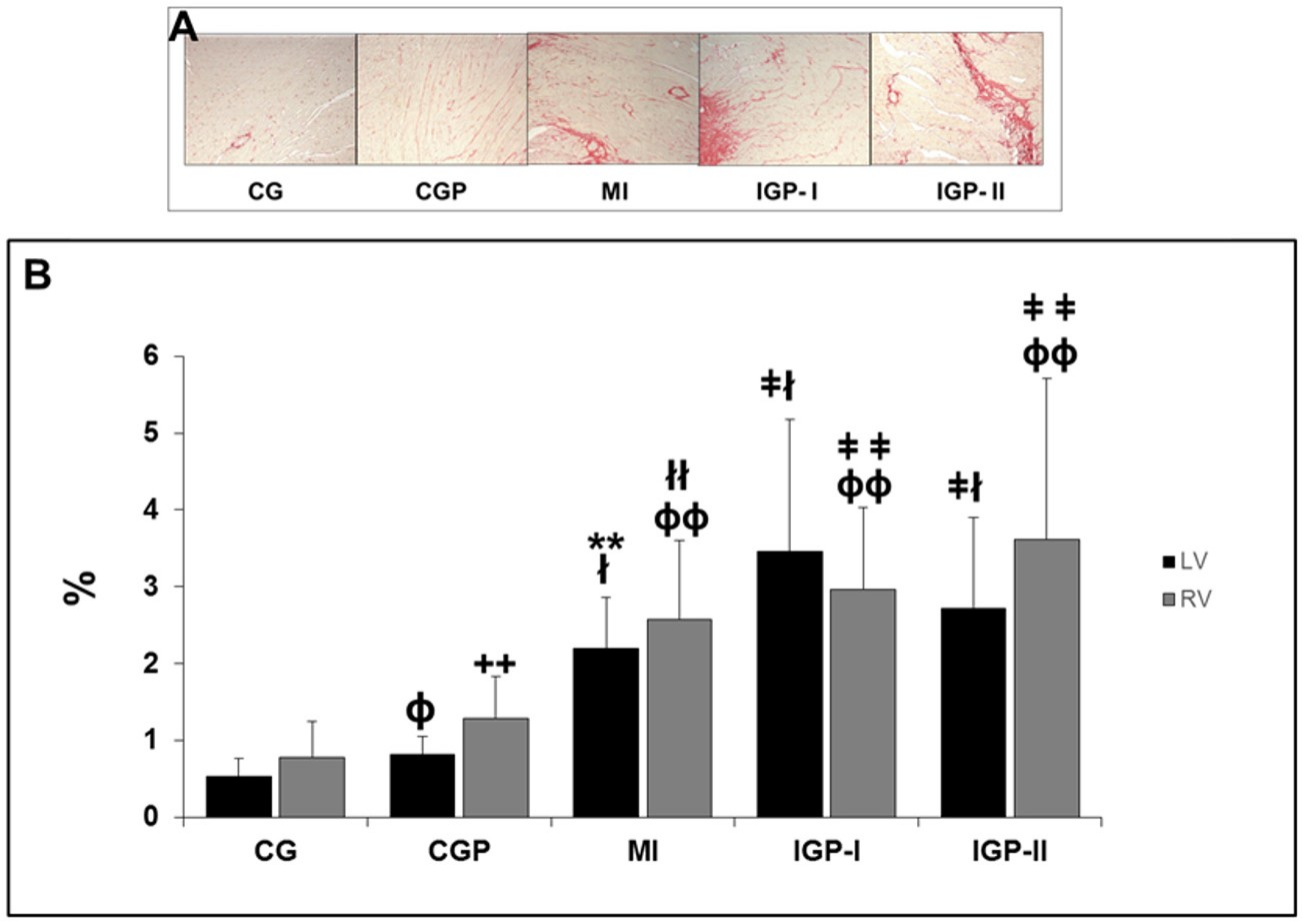
Morphometry. **A:** Picrosirius red-stained tissue showing in red the collagen deposition in the myocardium of the Control group (CG), Control group exposed to PM_2.5_ (CGP), Myocardial infarcted group (MI), Infarcted group immediately exposed to PM_2.5_ (IGP-I), Infarcted group previously exposed to PM_2.5_ (IGP-II). **B:** Left ventricular-interstitial collagen volume fraction (LV-ICVF) ^ɸ^p = 0.002 (CG [15] *vs* CGP [15]), ^ł^p<0.0001 (CG [15] *vs* MI [7], IGP-I [11], and IGP-II [11]), **p<0.0001 (CGP *vs* MI) and ^ǂ^p = <0.01(CGP *vs* IGP-I and IGP-II). Right ventricle interstitial collagen volume fraction (RV-ICVF). ^++^p<0.021 (CG *vs* CGP), ^ɸɸ^p<0.0001 (CG *vs* MI, IGP-I and IGP-II), ^łł^p = 0.0006 (CGP *vs* MI) and ^ǂǂ^p<0.001(CGP, IGP-I and IGP-II).

LV-ICVF (left ventricular-interstitial collagen volume fraction) increased in all groups compared with that in controls (p≤0.001). We should point out that CGP had higher LV-ICVF than CG had (p = 0.002). Similarly, results were observed in the right ventricle (RV) with increased RV-ICVF in all groups compared with CG. However, we did not observe an increase in ICVF in the LV or RV, because of PM_2.5_ in MI groups (Fig 3A and 3B).

**Fig 4:**
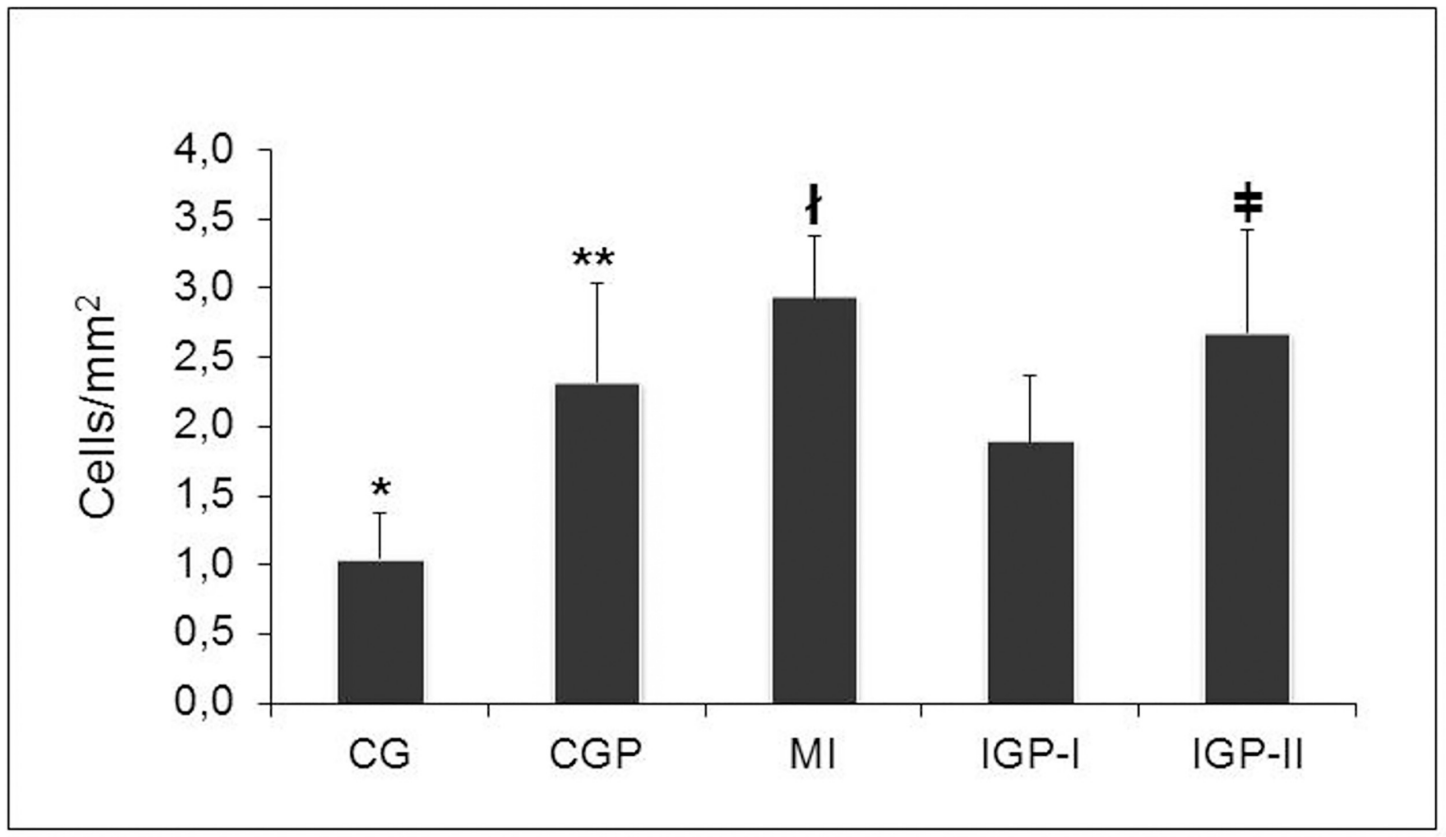
Inflammatory cell presence. *p<0.0001 (CG vs CGP, MI, IGP-I, and IGP-II, **p<0.05 (CGP vs MI, IGP-I, and IGP-II) ^**ł**^p<0.001 (MI vs IGP-I and IGP-II) and ^ǂ^ p<0.001 (IGP-I vs IGP-II). Control group (CG), Control group exposed to PM_2.5_ (CGP), Myocardial infarcted group (MI), Infarcted group immediately exposed to PM_2.5_ (IGP-I), Infarcted group previously exposed to PM_2.5_ (IGP-II).

We found that all study groups had greater inflammatory cell infiltration compared with CG, including CGP (p<0.0001). However, PM_2.5_ exposure did not increase inflammatory cell infiltration of infarcted groups. In fact, the MI had more cells than exposed groups had (p<0.001). Again, IGP-II had more inflammatory cells than IGP-I had (p<0.001) (Fig 4).

### Infarct size

Infarction size was not different in the infarcted groups whether exposed or not to PM_2.5_. In fact, air pollution did not affect the size of the infarcted area (MI = 27.30 ± 10.09%, IGP-I = 24.50 ± 8.79%, and IGP-II = 27.90 ± 11.97%; p = 0.660).

### Echocardiography

**Fig 5:**
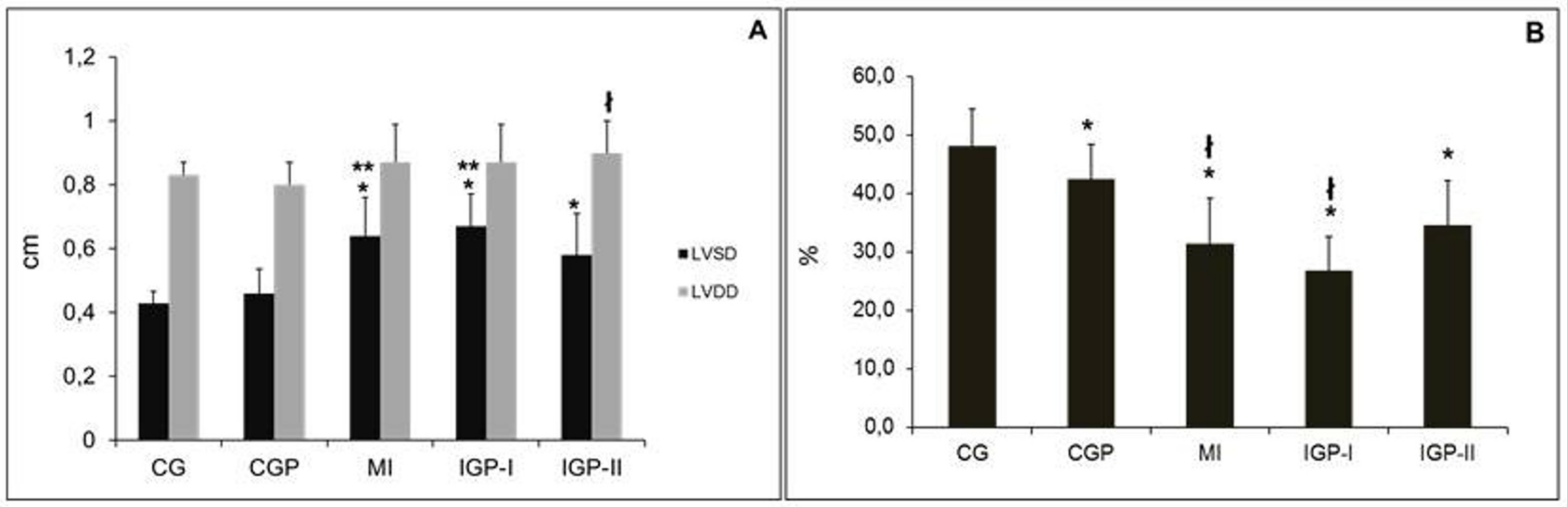
Echocardiography. **A:** Left Ventricular Dimension. Left Ventricular Systolic Diameter (LVSD) *p≤0.003 (CG [15] vs MI [8], IGP-I [11], and IGP-II [9]), **p≤0.018 (CGP [15] vs MI, and IGP-I) and Left Ventricular Diastolic Diameter (LVDD)—^ł^p = 0.020 (CGP vs IGP-II) (n = 58). **B:** Left Ventricular Function. Fractional Shortening (FS%) *p≤0.003 (CG *vs* CGP, MI, IGP-I, and IGP-II) and ^ł^p≤0.05 (CGP *vs* MI and IGP-I). Control group (CG), Control group exposed to PM_2.5_ (CGP), Myocardial infarcted group (MI), Infarcted group immediately exposed to PM_2.5_ (IGP-I), Infarcted group previously exposed to PM_2.5_ (IGP-II).

The infarcted groups had a larger left ventricular systolic diameter (LVSD) than controls had (p≤0.003). Also in the IGP-II group, the left ventricular diastolic diameter (LVDD) was larger than that in the CGP group (p≤0.002). However, PM_2.5_ did not increase this enlargement in the infarcted groups (Fig 5A). Regarding left ventricular function, all groups, including CGP, had lower fractional shortening compared with controls. Once again, PM_2.5_ did not increase ventricular impairment. We should point out again that CGP had lower fractional shortening (FS) than CG had (Fig 5B).

### Gene expression

PM_2.5_ exposure increased brain natriuretic peptide (BNP) expression in CGP and infarcted groups compared with controls. BNP was even higher in the IGP II group, which was the group with the greatest exposure time. However, this result was not statistically significant (CG = 0.16 ± 0.21, CGP = 0.28 ± 0.61, MI = 0.20 ± 0.10, IGP-I = 0.16 ± 0.20, and IGP-II = 0.73 ± 1.68; p = 0.118).

**Fig 6:**
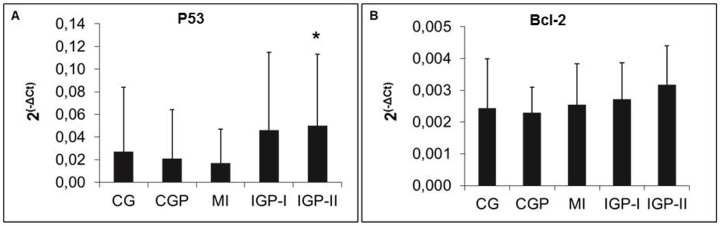
Apoptosis. **A:** Absolute expression of p53 (*p = 0.020) (CGP *vs* IGP-II) (n = 60). **B:** Absolute expression of Bcl-2 (p = 0.27) (n = 60). Control group (CG), Control group exposed to PM_2.5_ (CGP), Myocardial infarcted group (MI), Infarcted group immediately exposed to PM_2.5_ (IGP-I), Infarcted group previously exposed to PM_2.5_ (IGP-II).

Evaluation of apoptosis using the p53 gene was expressed more in the IGP-I (p = 0.52) and IGP-II (p = 0.12) groups than in the control group, but with no statistical difference. However, the IGP-II (p = 0.02) compared with the CGP had a higher expression of the p53 gene. The Bcl-2 gene was not significantly different between groups (p = 0.27) (Fig 6A and 6B).

**Fig 7:**
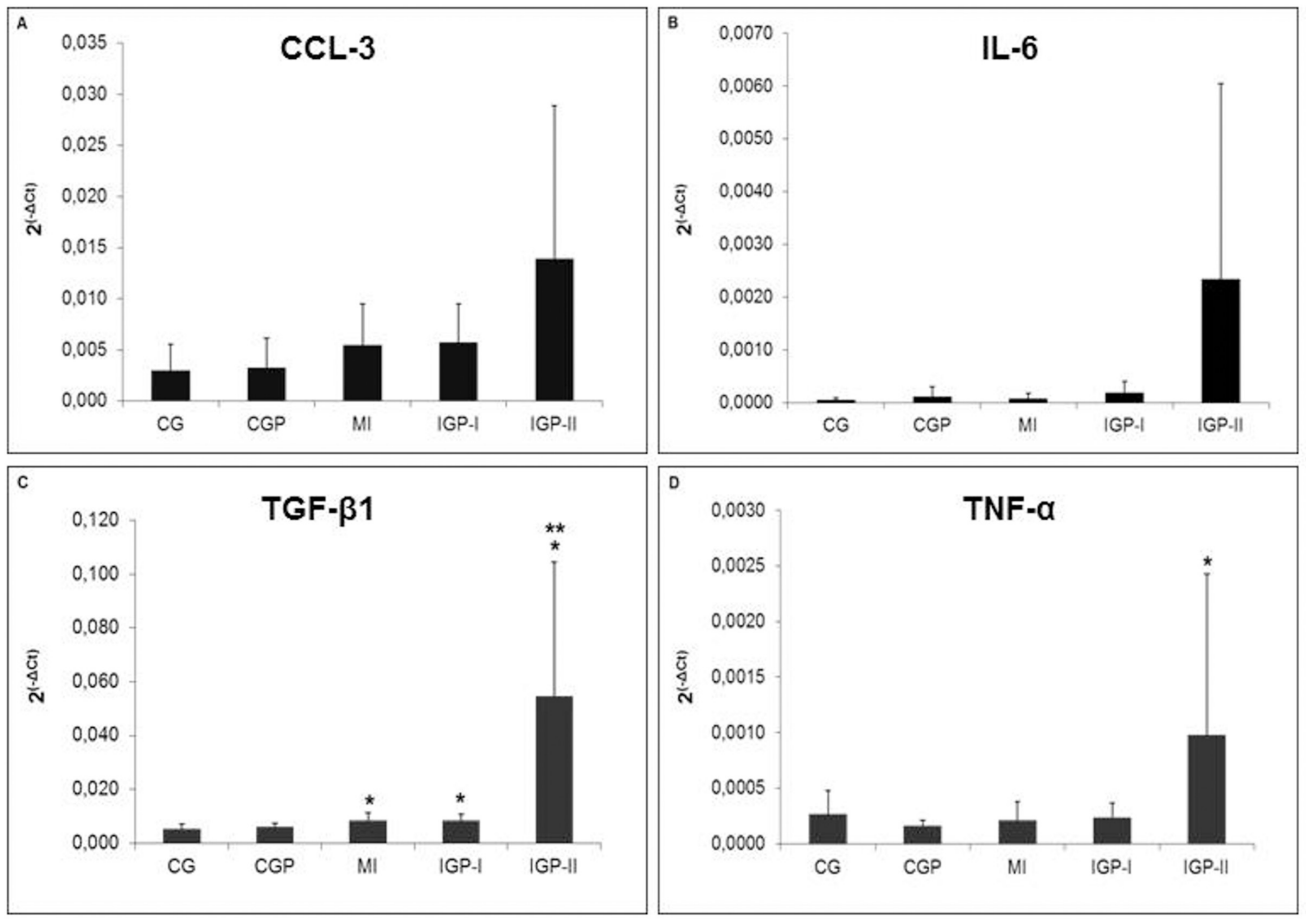
Inflammatory profile. **A:** Absolute expression of CCL-3 (p = 0.15) (n = 55). **B:** Absolute expression of IL-6 (p = 0.07) (n = 60). **C:** Absolute expression of TGF-β1 *≤0.04 (CG *vs* MI, IGP-I and IGP-II) and **p = 0.001 (CG *vs* IGP-II) (n = 60). **D:** Absolute expression of TNF-α *p = 0.012 (CGP *vs* IGP-II) (n = 60). Control group (CG), Control group exposed to PM_2.5_ (CGP), Myocardial infarcted group (MI), Infarcted group immediately exposed to PM_2.5_ (IGP-I), Infarcted group previously exposed to PM_2.5_ (IGP-II).

**Fig 8:**
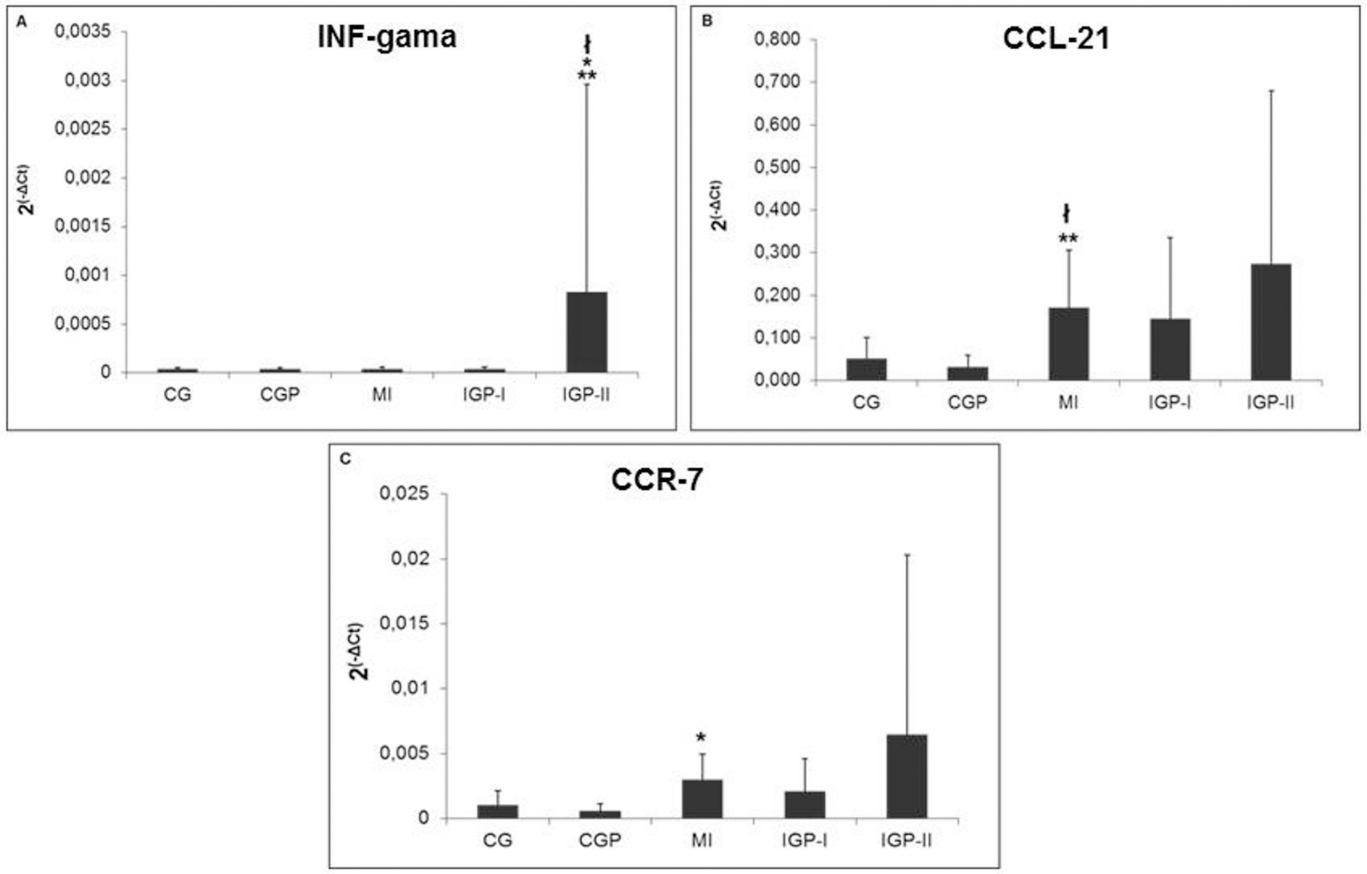
Inflammatory profile. **A:** Absolute expression of INF-gamma (*p = 0.007) (CG *vs* IGP-II), (**p = 0.002) (CGP *vs* IGP-II) and (^ł^p≤0.01) (IGP-II *vs* MI and IGP-I) (n = 59). **B:** Absolute expression of CCL-21 (^ł^p = 0.04) (CG *vs* MI) and (**p = 0.004) (CGP *vs* MI) (n = 55). **C:** Absolute expression of CCR-7 (*p = 0.003) (CGP *vs* MI) (n = 55). Control group (CG), Control group exposed to PM_2.5_ (CGP), Myocardial infarcted group (MI), Infarcted group immediately exposed to PM_2.5_ (IGP-I), Infarcted group previously exposed to PM_2.5_ (IGP-II).

In analyzing an inflammatory profile, we obtained the results shown in Figs 6 and 7. Despite the fact that genes (CCL-3 and IL-6) (Fig 7A and 7B) are expressed more in IGP-II, there were no significant differences (p = 0.15 and p = 0.07, respectively). We observed statistical differences between groups in TGF-β1 (CG *vs* MI and IGP-I, p≤0.04 and CG *vs* IGP-II, p = 0.001) (Fig 7C) and TNF-α (CGP *vs* IGP-II, p = 0.012) (Fig 7D). INF-gamma was higher in IGP-II than in all the other groups (p<0.01) (Fig 8A), CCL-21 (CG *vs* MI, p = 0.04) (Fig 8B) and (CGP *vs* MI, p = 0.004); CCR-7 (CGP *vs* MI, p = 0.003) (Fig 8C). TGF-β1 expression was higher in infarcted groups compared with CG (p≤0.04) and even higher in IGP-II (p = 0.001). TNF-α was higher in IGP-II (p = 0.01). We observed that in all evaluated inflammatory cytokines the profile was the same with increased expression in IGP-II compared with that in controls or with infarcted groups even with no statistical differences in some of them.

### Oxidative stress

#### ADMA

Despite ADMA being considered an important marker of cardiovascular disease, we found no differences in ADMA between groups (CG = 0.27 ± 0.10 μmol/L; CGP = 0.21 ± 0.05 μmol/L; MI = 0.22 ± 0.05 μmol/L; IGP-I = 0.22 ± 0.05 μmol/L; and IGP-II = 0.22 ± 0.07 μmol/L; p = 0.170).

#### Glutathione

We observed a higher concentration of GSH in CGP and infarcted groups compared with CG (CG = 4.18 ± 2.36μM; CGP = 6.19 ± 1.94μM; MI = 7.10 ± 1.83μM; IGP-I = 8.06 ± 3.45μM; and IGP-II = 7.86 ± 6.53μM; p≤0.03).

## Discussion

Inflammation, oxidative stress, and apoptosis are the pathways involved in collagen deposition in the myocardium, inducing structural, geometric, and functional remodeling. Epidemiological studies have confirmed an association of PM_2.5_ with cardiovascular morbidity and mortality with increasing hospital admissions. Increasing pollution in the short term over a period of hours during a day may cause cardiac death, myocardial infarction, and intensify heart failure [3]. We produced a real-life scenario of exposure to air pollution with the accumulated dose of 600/800 μg/m^3^ daily [2, 20]. This concentration is equivalent to that which a person can be exposed to on a daily basis in large urban areas. In the present study, we observed that pollution stimulated collagen accumulation in the myocardial interstitial space even in healthy hearts. This collagen accumulation was identified by the presence of LV-ICVF, which was approximately 2 times higher in the CGP than in the CG group. This has already been reported in a study conducted in 2012 [20]; however, the animals in that study experienced a longer period of exposure (9 months) under a much lower concentration of particulate matter than that used in the present study. We also quantified the collagen accumulation in the right ventricle separately and also showed that collagen deposition was 2 times higher in the CGP group than in the CG group. This approach, measuring left and right ventricles separately, is of fundamental importance, because it allows identification of the effects of PM_2.5_ on the heart independent of any impairment in the pulmonary territory. Because we know that PM may cause pulmonary injury, including inflammation, cell loss, and augmentation of vascular resistance, PM could also have an influence on the right ventricle. Therefore, separate measurement of the left ventricle confirmed the direct influence of PM_2.5_ on the heart. As expected, myocardial infarction promoted intense fibrosis in the left and right ventricles and in the remote uninjured areas. These data were expected, as seen in the early study by Frimm C, et al. [21], which demonstrated an intense collagen deposition in the infarcted and remote areas of MI. Several signaling pathways through the syncytial interstitial space and cell-to-cell modulation propagate the fibrotic response. The cascades of myocardial injury by activated pathways are sometimes coincidental to those activated by PM in different tissues. It would, therefore, be expected that in hearts injured because of MI, PM_2.5_ would amplify the scaring response in the myocardium. However, we did not observe this exacerbation of collagen accumulation in the myocardial interstitial space stimulated by PM_2.5_ in the infarcted groups as shown by LV and RV-ICVF.

The MI size also corroborated this finding based on the lack of an increased infarcted area by PM_2.5_ exposure. On the other hand, Cozzi E et al. [22] found an enlargement of the MI size in animals exposed to PM. However, they studied ultrafine particle exposure in acute myocardial injury by using an ischemic/reperfusion model. Two hypotheses can be suggested in this scenario. The first is that the cascade of mediators acutely activated by ischemic injury is much more intense than that stimulated by pollution overlapping this insult regarding myocardial fibrosis in the chronic phase. It includes neurohumoral activation as the case of renin angiotensin aldosterone and sympathetic nervous systems and inflammatory and oxidative stress pathways. These mediators, however, were able to significantly increase ICVF in healthy hearts. Another hypothesis is that the more prolonged exposure to pollution could intensify or perpetuate the activation of the cascade of inflammation and oxidative stress and, therefore, the modulation of fibrosis. This speculation is reinforced when we look at RV-ICVF that, although not statistically significant, is approximately 25% higher in the IGP-II group (longer PM_2.5_ exposure) than in the MI group. Moreover, the entire inflammatory profile was higher in the IGP-II group, and some aspects were statistically different. Similarly, the study by Wold et al. [20] reported that despite a much smaller dose of exposure to PM, the 9-month period was enough to cause myocardial fibrosis. The exposure to fine particulate matter leads to microvascular inflammation, thrombosis, and systemic endothelial changes, resulting in decreased contractility [22]. In this scenario, inflammatory mediators represent a key factor in myocardial injury. We know that the innate inflammation response is a protective mechanism. However, the levels and the persistence of the inflammatory mediators promote the loss of cardiomyocytes, either by necrosis or apoptosis. In the present study, we observed significant stimulation by PM_2.5_ of inflammatory pathways. This stimulus was most evident in animals from the IGP-II group that were exposed to PM_2.5_ for 7 weeks. TGF-β expression, TNF-α, and IFN-gamma were statistically significant in this context. Levels of other molecular markers analyzed, such as the cytokine expression of CCL-3, IL-6, CCL-21, and CCr-7, were also higher in IGP-II but did not reach significant values. High levels of cytokines stimulated by PM_2.5_ have been demonstrated in other studies [8, 23–25]. However, the relationship between inflammation and fibrosis, specifically in the myocardium, was not clear. We note that in the IGP-II group the average levels of inflammatory mediators were higher, coinciding with the higher deposition of collagen in the myocardium, mainly in the RV. In this group, however, although PM_2.5_ was observed repeatedly and consistently, it in fact did not exacerbate the inflammatory response after ischemic myocardial injury, except INF-gamma, which was higher in the IGP-II group than in the MI group. A complex cascade of events is started by pollution causing an interchange response between inflammatory and oxidative stress pathways. Our findings corroborate the findings of other studies, because we observed an increase in serum glutathione in the animals exposed to PM_2.5_ [26, 27]. This can be confirmed by the significantly higher values in the healthy group exposed to PM_2.5_ (CGP) compared with CG. Yet regarding oxidative stress, once again, we observed high levels of glutathione in infarcted groups and even more in those exposed to PM_2.5_ (IGP-I and IGP-II), but without statistical significance. Considering the inflammation cascade, oxidative stress, and myocardial fibrosis [23, 28–30], the data are aligned with their standard appearance. Because of the inflammation and oxidative stress triggered by both ischemic injuries and/or by exposure to pollution, loss of cardiomyocytes occurred with subsequent replacement of collagen, leading to reparative fibrosis. It is well known that this loss of cell contractility occurs either because of necrosis or apoptosis. Our findings show an increase in p53, a pro-apoptotic mediator, especially in the infarcted groups exposed to PM_2.5_, more so in IGP-II. Nichols et al. [31], who demonstrated increased cardiomyocyte apoptosis in an acute PM exposure and using a specific PM, have already observed this phenomenon. However, in our study although higher in the infarcted groups exposed to PM_2.5_, levels of P53 did not reach significance compared with levels in the MI group. Regardless of the mechanism responsible for cell loss and myocardial structural remodeling, loss of systolic and diastolic function occurs, depending on the extent of cell damage, intracellular molecular behavior, and myocardial interstitial space. As might be expected, the infarcted groups had the most compromised systolic function compared with the control groups. The interstitial space was compromised, because collagen deposition had a pivotal role in this scenario of systolic function. This might be because, as we demonstrated, the infarcted areas were similar in all infarcted groups and even smaller in the infarcted group exposed to PM_2.5_ (IGP-I). However, IGP-I had the highest percentage of collagen in the interstitial remote area in the left ventricle and was also the group with lower fractional shortening between groups. This suggests that, in fact, a distortion of myocardial interstitial space is an important modulator of functional impairment of the heart. We should mention that PM_2.5_ did not cause greater loss of ventricular systolic function in the infarcted groups. However, PM_2.5_ did cause increased collagen deposition in the myocardial interstitial space in healthy hearts. It also promoted loss of systolic function in the hearts of the animals in the CGP group, thus confirming the importance of myocardial structural remodeling in cardiac function and the role of PM_2.5_ in structural and functional remodeling. Nichols et al. [31] using an acute PM exposure model has already demonstrated this loss of contractility.

We conclude that, in fact, PM_2.5_ plays a key role in structural myocardial remodeling resulting in the loss of heart function. However, air pollution did not increase this response in hearts injured by ischemia. Inflammation, oxidative stress, and apoptosis pathways appear to be involved directly in this process. Thus, this study defines another aspect of the damage to health caused by PM_2.5_, more specifically upon heart remodeling. These data added to those of other studies demonstrate the immediate need to develop policy measures for the control of PM_2.5_ in our environment.
